# The Prisoners of War Fund and the Hospitals

**Published:** 1920-03-20

**Authors:** 


### The Prisoners of War Fund and the Hospitals.

MR. F. R. C. PAYNE AND THE FUTURE.

The Leicester, Leicestershire, and Rutland Prisoners of War Fund Committee has issued an interesting and final report of the work well done and help rendered to local soldiers attached not only to the county regiments, but to any other of His Majesty's Forces. The total number of parcels despatched numbered over 200,000, and the committee reports that at least 90 per cent. of the beneficiaries reported that parcels reached them regularly, provided the men were in German camps and not kept behind the lines. The initiator of the great service thus rendered was the Rev. F. R. C. Payne, M.A., O.B.E., Vicar of St. Margaret's. What his church did was subsequently developed by means of systematic collections from all places of worship, and when the collection and packing and despatch of the parcels became too great for any one section the work was taken in hand by a representative committee, who organised private contributions and systematic collections in factories and workshops. At a later date the borough and county, while continuing to work jointly in the sending of parcels, organised separate funds. The work and responsibility were heavy, but so generous was the response that receipts amounting to £76,750 are recorded. Of this huge sum £47,635 resulted from general donations, £10,000 from the County Fund raised by the then High Sheriff, Mr. T. Fielding Johnson, Junr., £7,553 from men's relatives, £4,923 from the churches, £4,490 from workpeople, £1,393 from retail traders, and £754 from bank interest. Payments for food, etc., are scheduled at £60,914, so that £15,835 remained in hand. This balance enabled the committee to continue their public-spirited work by making grants to local charities. £10,000 was granted to the Leicester Royal Infirmary—timely aid in view of the huge programme of enlargement already foreshadowed in The Hospital; £1,000 each to the Maternity Hospital, the Institution for the Blind, the District Nursing Association, and the Leicester and County Saturday Hospital Society; and £500 each to other bodies. A highly successful and worthy service thus closes with timely help to local institutions, an earnest, let us hope, for future co-operation on voluntary lines, for the movement must not be allowed to die. What is being done to perpetuate it?

